# Molecular mechanisms of hotspot variants in cytoskeletal β‐actin associated with Baraitser–Winter syndrome

**DOI:** 10.1111/febs.70018

**Published:** 2025-02-10

**Authors:** Johannes N. Greve, Dietmar J. Manstein

**Affiliations:** ^1^ Institute for Biophysical Chemistry Hannover Medical School, Fritz–Hartmann Centre for Medical Research Germany; ^2^ Division for Structural Biochemistry Hannover Medical School Germany; ^3^ RESiST, Cluster of Excellence 2155 Hannover Medical School Germany

**Keywords:** actin, actinopathy, Arp2/3 complex, genetic disease, myosin

## Abstract

Baraitser–Winter cerebrofrontofacial syndrome (BWCFF) is the most common and best‐defined clinical entity associated with heterozygous single‐point missense mutations in cytoskeletal β‐actin. Patients present with distinct craniofacial anomalies and neurodevelopmental disabilities of variable severity. To date, the most frequently observed variants affect residue R196 of cytoskeletal β‐actin. Patients carrying the p.R196 variants are likely to suffer from pachygyria, probably due to neuronal migration defects contributing to the development of abnormal convolutions of the cerebral cortex. Here, we report on the recombinant production, purification and characterization of the BWCFF hotspot variants p.R196H, p.R196C and p.R196S. Our findings reveal that the stability of the monomeric variants remains unaffected, suggesting that the disease mechanism involves the incorporation of these variants into actin filaments. This incorporation alters F‐actin stability and polymerization dynamics to varying degrees, depending on the specific variant. These effects are consistent with the positioning of residue R196 near the helical filament axis. Observed changes include an increased critical concentration for polymerization, reduced elongation rates and accelerated filament depolymerization. Within the actin‐related protein 2/3 (Arp2/3)‐generated branch junction complex, which is critical for processes such as cell migration and endocytosis, residue R196 is located at the interface between the first protomer of the nucleated filament and the Arp2 subunit. Variant p.R196H specifically results in reduced branching efficiency and impaired branch stability. Future research will seek to elucidate the impact of these actin filament defects on cellular processes and their contribution to the multifaceted pathophysiology of BWCFF, with a particular emphasis on cortical development.

## Graphical abstract

Variants at position R196 in cytoskeletal β‐actin are the most common variants associated with Baraitser–Winter cerebrofrontofacial syndrome and result in craniofacial anomalies and neurodevelopmental disorders, most likely due to neuronal migration defects. This study explores the molecular mechanisms of p.R196 variants using purified proteins. The mutant proteins are stably produced and integrate into linear and branched actin networks, but severely perturb network turnover and integrity by destabilizing actin–actin and actin–Arp2/3 interactions.

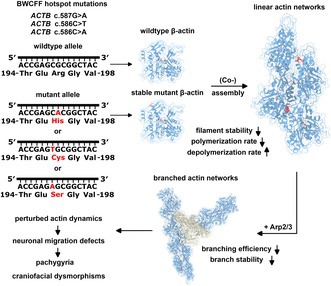

**Abbreviations**
ABP: actin‐binding protein
ADP: adenosine 5′‐diphosphate
Arp2/3: actin‐related protein 2/3
ATP: adenosine 5′‐triphosphate
BWCFF: Baraitser–Winter cerebrofrontofacial syndrome
c_c_
: critical concentration
DSF: differential scanning fluorimetry
DTT: 1,4‐dithiothreitol
EGTA: ethylene glycol‐bis(β‐aminoethyl ether)‐*N*,*N*,*N*′,*N*′‐tetra‐acetic acid
F‐actin: filamentous actin
FWHM: full width at half maximum
G‐actin: globular actin
GST: glutathione *S*‐transferase
HMM: heavy meromyosin
IC_50_
: half‐maximal inhibitory concentration
MOPS: 3‐(*N*‐morpholino)–propane–sulfonic acid
Myl12b: myosin light chain 12b
Myl6: myosin light chain 6
Myo5: myosin‐5A
NM2A: non‐muscle myosin‐2A
NPF: nucleation‐promoting factor
N‐WASP: neural Wiskott–Aldrich syndrome protein
PMSF: phenylmethylsulfonyl fluoride
Sf9: *Spodoptera frugiperda*‐9
TAME: tosyl‐l‐arginine methyl ester
TIRFM: total internal reflection fluorescence microscopy
T_M_
: thermal denaturation temperature
TPCK: tosyl‐phenylalanyl‐chlormethyl ketone
TRIS: tris(hydroxymethyl)aminomethane
TRITC: tetramethylrhodamine isothiocyanate
VCA: verprolin homology domain (V), cofilin homology domain (C) and acidic (A)
WT: wild‐type
ε‐ATP: 1,N^6^‐ethenoadenosine 5′‐triphosphate

## Introduction

Cytoplasmic actin filaments are composed of the ubiquitous actin isoforms cytoskeletal β‐ and γ‐actin, encoded by *ACTB* and *ACTG1* [1]. Essential cellular processes like cell migration, adhesion, division and signal transduction depend on an intact actin cytoskeleton and a robust remodelling of cytoskeletal actin structures, which enables cells to respond to intra‐ and extracellular stimuli by conferring cytoskeletal plasticity [2, 3, 4]. Actin remodelling, involving assembly and disassembly of actin filaments and higher‐order F‐actin structures, is tightly regulated by a large number of G‐ and F‐actin binding proteins. This regulation allows spatio‐temporal control of cellular actin dynamics and drives functional compartmentalization of the actin cytoskeleton [5, 6].

Heterozygous missense mutations in *ACTB* or *ACTG1* result in a broad spectrum of non‐muscle actinopathy phenotypes observed in affected patients [7, 8, 9, 10, 11]. To date, the best described clinical entities associated with missense variants in *ACTB* and *ACTG1* are non‐syndromic hearing loss due to missense mutations in *ACTG1* [12, 13, 14], and the Baraitser–Winter cerebrofrontofacial syndrome (BWCFF) caused by missense variants in *ACTB* and *ACTG1* [7, 8, 15]. BWCFF is associated with brain malformations and neuronal migration defects that lead to neurodevelopmental disorders and intellectual disabilities. The patients present with distinct craniofacial features such as facial malformations, microcephaly, ptosis, hypertelorism and ocular coloboma syndrome [7, 8]. The more severe cases of BWCFF are regularly observed in patients carrying variants in *ACTB* [10]. Missense variants associated with BWCFF are scattered throughout the *ACTB* and *ACTG1* sequences, with no apparent preference for any particular region. Nonetheless, specific variants are more often observed in affected patients and are thus termed hotspot variants. These include the p.R196H variant in cytoskeletal β‐actin, which is by far the most frequently observed variant among all BWCFF variants. It causes a prototypical BWCFF phenotype characterized by typical BWCFF‐specific craniofacial anomalies and developmental delay due to cerebral cortex malformations [8]. Patients present with moderate‐to‐severe intellectual disability and delayed speech. Two other variants p.R196S and p.R196C have been observed at this position, both resulting in a similar phenotype. Additional central nervous system disorders observed in some patients, such as muscular hypotonia and spasticity, have been interpreted to be patient specific rather than variant specific [7, 8].

Due to the large number of possible disease mechanisms, the link between the observed clinical phenotype in the patient and the perturbation of the molecular mechanisms of actin dynamics caused by the mutant actin remains a major challenge in the field of actin‐associated diseases. Postulated disease mechanisms include functional haploinsufficiency due to instability and rapid degradation of the mutated actin [16], distorted regulation of cellular actin dynamics due to direct or allosteric perturbances of actin–actin and actin–ABP interaction interfaces [17, 18, 19, 20] and formation of toxic rod‐like actin aggregates [21].

**Fig. 1:**
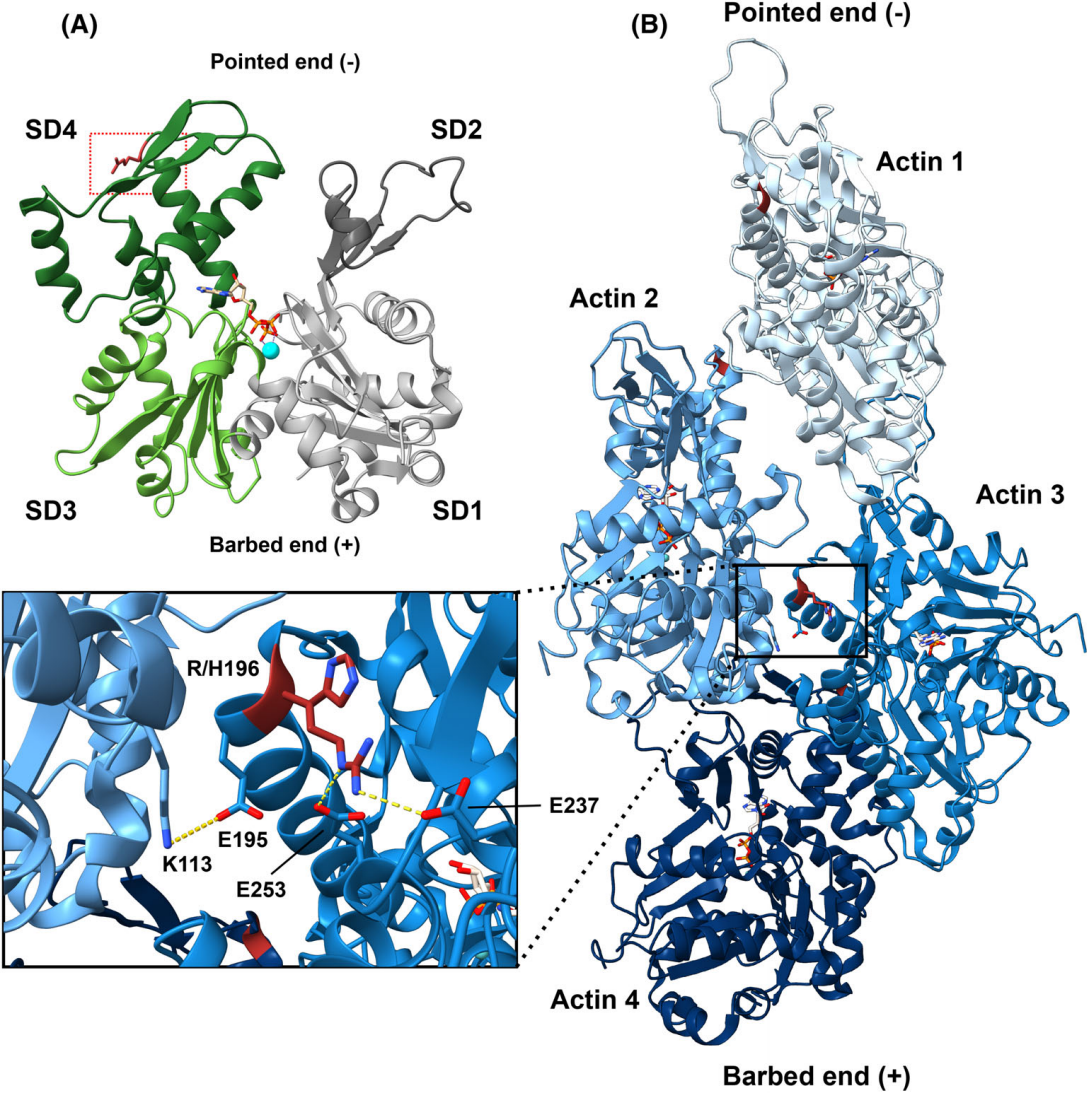
Location of the mutated residue R196 in the G‐ and the F‐actin structure. (A) Structural model of cytoskeletal β‐actin (based on PDB‐ID: 2BTF [63]). Subdomains (SDs) of actin are coloured in grey (SD1), dark grey (SD2), light green (SD3) and green (SD4). ATP is bound to the nucleotide‐binding site; the coordinated Mg^2+^ ion is depicted in cyan. The mutated residue R196 is located in SD4 and coloured red. Structure visualization was performed using chimerax [62]. (B) Structural model of the human β‐actin filament in the Mg^2+^‐ADP‐bound state (PDB‐ID: 8DNH [64]). Actin protomers are shown in different shades of blue. ADP is coloured light grey, and the residue R196 is coloured red. The zoom‐in shows the position of residue R196 and the variant residue H196 close to the longitudinal axis of the filament (for visualization of variant residues C196 and S196, see Fig. S1). Salt bridges established between R196 and E237 and E253 in the same actin protomer are indicated by yellow dotted lines. The salt bridge formed by the neighbouring residue E195 and residue K113 in the protomer of the opposing filament strand is shown by the yellow dotted line (H‐bond cut‐off: 4 Å). Structure visualization was performed using chimerax [62].

Here, we report the biochemical characterization of the BWCFF hotspot variants p.R196H, p.R196C and p.R196S in cytoskeletal β‐actin. Residue R196 is located in a flexible loop in subdomain 4 of the actin protomer, away from the sensitive hinge region, the nucleotide‐binding cleft and the target‐binding cleft, which forms a major binding site for monomer‐binding ABPs (Fig. 1A). After integration of the monomer into a filament, R196 is found close to the helical axis of the actin filament. Here, the neighbouring residue E195 is involved in the formation of a salt bridge with K113 in the opposite protomer (Fig. 1B and Fig. S1). This interaction constitutes one of the few lateral contacts across the filament axis that is involved in filament stabilization. These lateral contacts are far fewer in number than the more extensive longitudinal contacts formed between the molecules in the individual filament strands [22]. Furthermore, R196 forms several electrostatic interactions with charged residues of the same actin protomer (Fig. 1B and Fig. S1).

We show that the hotspot mutations at position 196 strongly perturb actin assembly dynamics *in vitro* by increasing the critical concentration for actin polymerization, reducing the rate of filament elongation and increasing the rate of filament depolymerization. With respect to the interaction with key actin‐binding proteins, we show that defects in polymerization behaviour persist in the presence of the actin nucleator Arp2/3 complex. We demonstrate that specifically p.R196H interacts less efficiently with the Arp2/3 complex, resulting in fewer and less stable branched filaments. Furthermore, we provide evidence for myosin‐isoform‐specific changes in the ability of p.R196H filaments to function as myosin motor tracks.

## Results

### Production of cytoskeletal β‐actin and the p.R196 variants

**Fig. 2:**
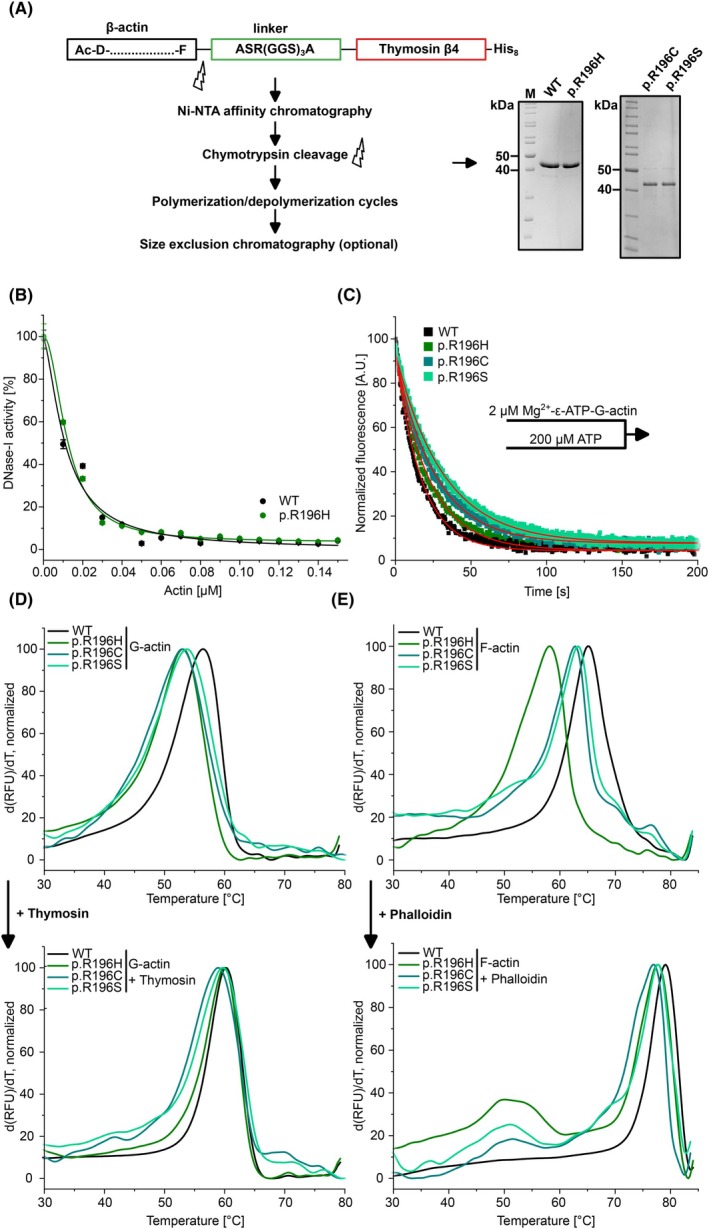
Analysis of actin folding, nucleotide exchange and thermal stability (A) Schematic representation of the strategy used to purify recombinant human cytoskeletal β‐actin wild‐type (WT) and p.R196 variants. The SDS gels show purified WT and p.R196 variant cytoskeletal β‐actin. (B) Inhibition of DNase‐I activity by monomeric p.R196H and β‐actin WT. Data are the mean of three individual experiments ± SD. A Hill equation was fitted to the data, which yields the half‐maximal inhibitory concentration (IC_50_). (C) The rate of nucleotide dissociation (*k*_−T_) was determined for monomeric variant and WT β‐actin using fluorescently labelled ATP (ε‐ATP). Shown are representative experimental traces to which single‐exponential functions (red) were fitted (*N* = 15 for WT, *N* = 15 for p.R196H, *N* = 11 for p.R196C and *N* = 9 for p.R196S). (D, E) The protein denaturation temperature (*T*_M_) of monomeric p.R196 variant and WT actin in the absence (*N* = 6 for WT, *N* = 6 for p.R196H, *N* = 4 for p.R196C and *N* = 4 for p.R196S) and presence of thymosin β‐4 (*N* = 4 for WT, *N* = 4 for p.R196H, *N* = 4 for p.R196C and *N* = 4 for p.R196S) were determined by differential scanning fluorimetry (DSF) (D). *T*_M_ of filamentous p.R196 variant and WT actin in the absence (*N* = 12 for WT, *N* = 9 for p.R196H, *N* = 6 for p.R196C and *N* = 6 for p.R196S) and presence of phalloidin (*N* = 9 for WT, *N* = 9 for p.R196H, *N* = 6 for p.R196C and *N* = 6 for p.R196S) were determined using the same approach (E). Representative experimental traces are shown. The thermal denaturation temperature was derived from the peak of the melting curves.

We produced cytoskeletal β‐actin and the p.R196 variants as actin–thymosin‐β4 fusion proteins in the baculovirus/Sf9 insect cell expression system [23]. To facilitate purification and prevent polymerization during recombinant production and initial purification steps, the monomer sequestering protein thymosin‐β4 carrying a C‐terminal His_8_‐tag was fused to the β‐actin C terminus (Fig. 2A). Immobilized metal affinity chromatography was employed for the initial purification of the fusion protein, followed by chymotrypsin‐mediated cleavage to remove the C‐terminal linker and thymosin‐β4 moiety. This process yields recombinant cytoskeletal actin devoid of any non‐native residues and with correct N‐terminal acetylation of aspartate‐2 [24, 25]. The recombinant actin was purified further by cycles of polymerization and depolymerization. A typical preparation started with 2 × 10^9^ cells and yielded 6–8 mg of purified protein for WT cytoskeletal β‐actin and 3–5 mg of purified protein for the p.R196 variants (*N* = 3 preparations for WT β‐actin, *N* = 4 preparations for p.R196H and *N* = 1 preparations for p.R196C and p.R196S variants). The lower yields obtained with the p.R196 variants can be attributed to a reduced ability to form filaments during the final purification steps comprising polymerization and depolymerization cycles.

### Effect of mutation R196H on protein folding, nucleotide interaction and thermal stability

We initially assessed the effect of the R196H mutation on the folding of the protein using a DNase‐I inhibition assay (Fig. 2B). Misfolded or denatured actin shows a reduced capacity of DNase‐I inhibition, as previously shown [26]. We observed no significant change in the observed IC_50_ value of p.R196H compared to WT actin (IC_50,WT_ = 10.7 ± 1.1 nm; IC_50,R196H_ = 12.4 ± 0.6 nm).

Efficient nucleotide binding is important for the full functionality and stability of G‐actin. Therefore, we investigated the ability of monomeric variant and WT actin to interact with ATP by performing nucleotide dissociation experiments with fluorescent ε‐ATP using a stopped‐flow apparatus (Fig. 2C). To determine the dissociation rate *k*_−T_, we monitored the displacement of fluorescent ε‐ATP from the nucleotide‐binding site after mixing with excess unlabelled ATP. All variants showed a slight but significant, variant‐specific reduction in the observed ATP dissociation rate from the nucleotide‐binding site (*k*_−T,R196H_ = 0.051 ± 0.003 s^−1^, *k*_−T,R196C_ = 0.038 ± 0.002 s^−1^ and *k*_−T,R196S_ = 0.032 ± 0.001 s^−1^) compared to WT monomers (*k*_−T,WT_ = 0.065 ± 0.002 s^−1^).

Next, using differential scanning fluorimetry (DSF) [27], we analysed the thermal denaturation temperature (*T*_M_) of the monomeric (G–) form and filamentous (F–) forms of WT and variant actin (Fig. 2D,E). All mutations result in a slight decrease of approximately 3 °C in the *T*_M_ for mutant G‐actin (Fig. 2D, Table 1). The actin‐sequestering protein thymosin‐β4 binds a significant amount of G‐actin *in vivo* and it has previously been shown that binding of thymosin‐β4 to G‐actin significantly increases *T*_M_ compared to uncomplexed G‐actin [28]. Thus, we performed thermal denaturation experiments in the presence of thymosin‐β4. We found that the presence of thymosin‐β4 increased *T*_M_ for both WT and variant actin, offsetting to a large extent the observed difference in *T*_M_ between uncomplexed WT and variant G‐actin (Fig. 2D, Table 1). Next, we determined the *T*_M_ of WT and variant filaments. In accordance with previous studies [29], we found that compared to G‐actin, WT filaments show an increase in *T*_M_ of approximately 8.5 °C to 64.7 ± 0.5 °C. Filaments containing p.R196H show a smaller increase in *T*_M_ of 5.8 °C from 53.1 ± 0.1 to 58.9 ± 0.6 °C. The R196C and R196S mutations had a less pronounced impact on the thermal stability of filamentous actin compared to p.R196H, but still resulted in reduced stability compared to WT actin (Fig. 2E, Table 1). The more asymmetric peak shape obtained for the variant filaments, which partially overlaps with the melting curve obtained for the monomeric actin variants, is consistent with a disturbed G‐ to F‐actin ratio. DSF experiments with WT and variant F‐actin in the presence of the *Amanita phalloides* toxin phalloidin confirmed this finding (Fig. 2E). Phalloidin is commonly used in biochemical studies to produce stable filaments at concentrations below the critical concentration required for polymerization (≥ 0.1 μm). It binds to F‐actin with high affinity and stabilizes the filamentous form by increasing the number of lateral and longitudinal contacts and restricting the relative movement of the two strands of the F‐actin helix [30]. The stabilizing effect of phalloidin on WT filaments results in an increased *T*_M_ in the presence of phalloidin (*T*_M_ = 78.7 ± 0.5 °C) and an apparent reduction in the full width at half maximum (FWHM). We interpret this as an increase in cooperativity of the denaturation process due to reduced structural flexibility of the actin filament. In the case of variant filaments, two peaks were observed in the presence of phalloidin: a larger peak at approximately the same temperature as in the WT samples and a smaller peak with a maximum at approximately 52 °C, which matches the melting curve of uncomplexed variant G‐actin. These findings suggest that phalloidin is unable to fully shift the G‐actin to F‐actin ratio in the variant actin samples to the F‐form, stabilizing variant F‐actin less effectively than WT F‐actin.

**Table 1:** Nucleotide dissociation rates and thermal denaturation temperatures determined for WT and variant β‐actin.

Parameter	WT	p.R196H	p.R196C	p.R196S
IC_50_ (DNaseI) [nm]	10.7 ± 1.1	12.4 ± 0.6	n.d.	n.d.
*k*_−T_ (Mg^2+^‐ε‐ATP) [s^−1^]	0.065 ± 0.002	0.051 ± 0.003	0.038 ± 0.002	0.032 ± 0.001
*T*_M_ (Mg^2+^‐ATP‐G‐actin) [°C]	56.3 ± 1.1 60.3 ± 0.1 (+Tβ4)	53.1 ± 0.1 59.4 ± 0.6 (+Tβ4)	52.3 ± 0.4 58.7 ± 0.2 (+Tβ4)	54.1 ± 0.5 59.6 ± 0.2 (+Tβ4)
*T*_M_ (F‐actin) [°C]	64.7 ± 0.5 78.7 ± 0.5 (+Phall.)	58.9 ± 0.6 76.9 ± 0.5 (+Phall.)	61.8 ± 0.9 77.4 ± 0.4 (+Phall.)	62.9 ± 0.6 78.6 ± 0.6 (+Phall.)

### Mutations at position R196 result in perturbed polymerization and depolymerization dynamics

**Fig. 3:**
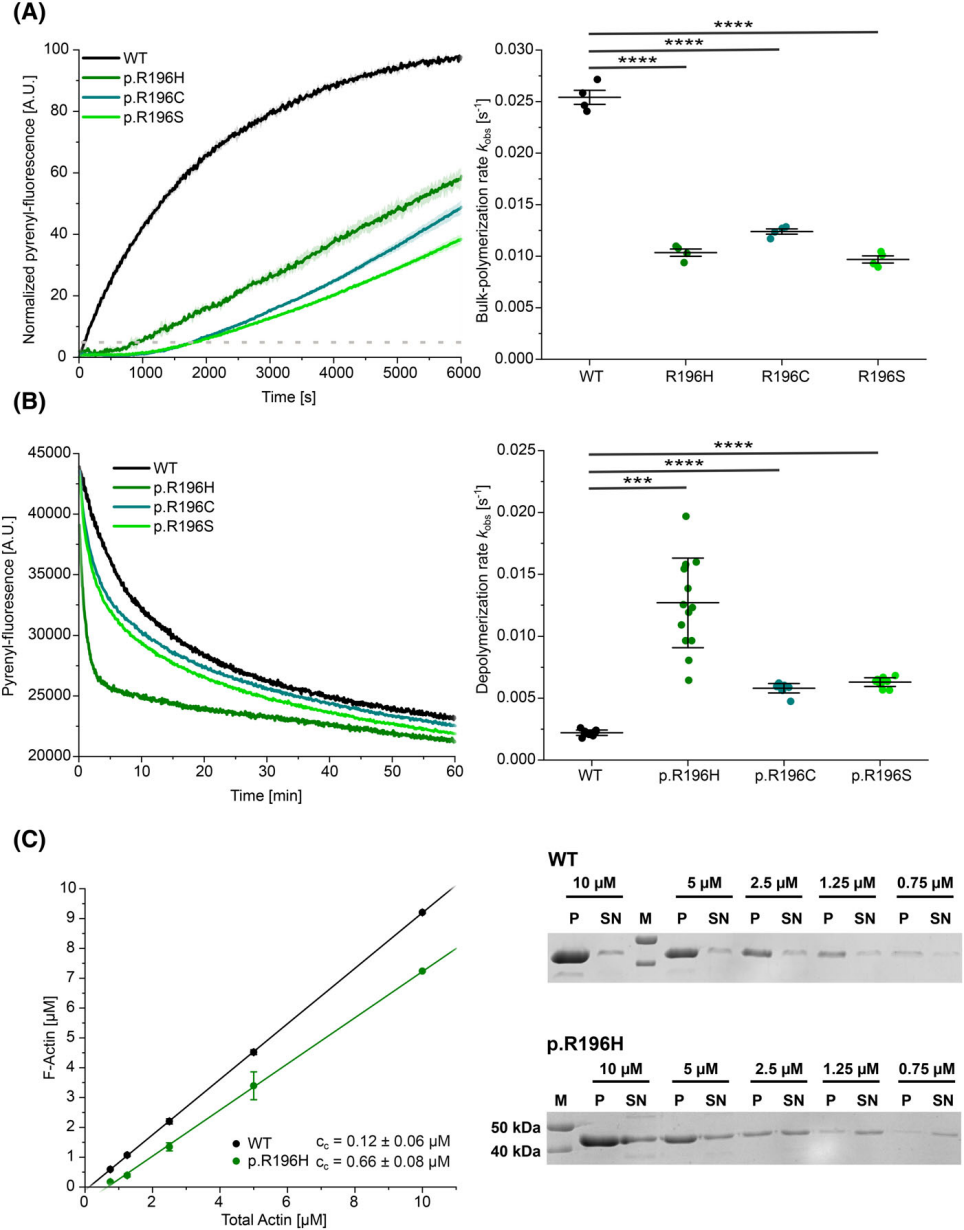
Analysis of the polymerization and depolymerization capacities of p.R196 variants and wild‐type β‐actin using pyrene‐labelled actin (A) Pyrene‐based polymerization experiment of 2 μm variant or wild‐type (WT) actin were performed using 5% pyrene‐labelled actin. (Left) The obtained traces from the polymerization experiments are shown as the mean ± SD (*N* = 4 for each protein). The dotted grey lines indicate 5% of the final pyrenyl fluorescence, the threshold that was used to determine the lag‐time *t*_lag_ in Table 2. (Right) Bulk polymerization rates determined from the experiments shown in (A). Every data point corresponds to an individual polymerization experiment (*N* = 4 for each protein). Individual values are summarized in Table 2. Data are shown as the mean ± SD. Statistical significance was determined by an unpaired two‐sample *t*‐test (**** ≙ *P* ≤ 0.0001). (B) Pyrene‐based dilution‐induced depolymerization experiments (5% pyrene‐labelled actin) were used to determine the apparent rate of filament depolymerization of variant and WT filaments. The depolymerization of filaments was induced by the addition of G‐buffer. (Left) Exemplary traces of individual depolymerization experiments performed with variant and WT actin. (*N* = 12 for WT, *N* = 14 for p.R196H, *N* = 12 for p.R196C and *N* = 12 for p.R196S.) (Right) Secondary plot summarizing the rates of filament depolymerization determined from all experiments. Every data point corresponds to an individual depolymerization experiment (*N* = 12 for WT, *N* = 14 for p.R196H, *N* = 12 for p.R196C and *N* = 12 for p.R196S). Data are shown as the mean ± SD. Statistical significance was determined by an unpaired two‐sample *t*‐test (*** ≙ *P* ≤ 0.001; **** ≙ *P* ≤ 0.0001). (C) High‐speed sedimentation experiments with varying actin concentrations were used to determine the critical concentration (c_c_) for polymerization of p.R196H and WT actin. (Left) The amount of F‐actin in the pellet fraction was determined by densitometry after SDS/PAGE. A linear regression was applied to the data, which yields the apparent critical concentration (c_c_) as the intercept with the *x*‐axis. Data are shown as the mean ± SD (*N* = 4 for each WT concentration and *N* = 3 for each p.R196H concentration except 10 μm where *N* = 1 due to the limited amount of purified protein) (Right) Exemplary SDS gel of sedimentation experiments performed with p.R196H and WT actin. m, Molecular weight marker.

We analysed the ability of monomeric variant actin to form filaments by means of pyrene actin‐based bulk polymerization assays (Fig. 3A). The interpretation of experimental data from pyrene‐based bulk polymerization studies is based on the hypothesis that the two phases of actin polymerization, nucleation and elongation, give rise to two distinct observable changes in pyrenyl fluorescence intensity over time. The first phase, characterized by a slow increase in pyrenyl fluorescence (the ‘lag–phase’), is primarily associated with the nucleation of actin filaments. This is followed by a second phase, marked by a more rapid increase in fluorescence, which reflects the rapid barbed‐end elongation of actin filaments. We determined the duration of the lag‐phase (*t*_lag_) and the bulk polymerization rate (*k*_obs_) by analysing the two phases in experiments performed with 2 μm variant or WT actin. We used 5% pyrene‐labelled WT or p.R196H actin for experiments performed with these proteins. For the p.R196C and p.R196S variants, we incorporated 5% pyrene‐labelled WT actin as a tracer to monitor the reaction progress. This approach was particularly necessary for the p.R196C variant, as the introduction of an additional cysteine residue renders specific labelling of cysteine‐374 unfeasible. Previous studies have demonstrated that using trace amounts of pyrene‐labelled WT actin does not obscure the mutation‐induced defects in polymerization dynamics [17, 31]. The three mutations decreased the bulk polymerization rate by approximately two‐ to threefold (Fig. 3A, Table 2). However, more substantial, variant‐specific effects were observed in the duration of the lag‐phase. The R196C and R196S mutations extended *t*_lag_ 22‐fold, whereas the R196H mutation caused an 11‐fold increase (Table 2).

**Table 2:** Rates determined from bulk polymerization and depolymerization studies using pyrene‐labelled actin.

Parameter	WT	p.R196H	p.R196C	p.R196S
Bulk‐polymerization rate *k*_obs_ [s^−1^]	0.0254 ± 0.0014	0.0103 ± 0.0007	0.0124 ± 0.0005	0.0097 ± 0.0007
Lag‐time *t*_lag_ [s]	80.0 ± 21.2	917.5 ± 92.1	1786.3 ± 49.5	1801.3 ± 60.5
Depolymerization rate *k*_obs_ [s^−1^]	0.0022 ± 0.0002	0.0127 ± 0.0036	0.0058 ± 0.0004	0.0063 ± 0.0004

To investigate the effect of the three variants on spontaneous filament disassembly, we performed dilution‐induced depolymerization experiments using pyrene‐labelled pre‐polymerized WT and variant actin (Fig. 3B). Consistent with reduced filament stability, we observed a 5.8‐fold increase in the depolymerization rate for p.R196H filaments compared to WT filaments. The p.R196C and p.R196S variants showed a less pronounced effect, resulting in approximately 2.7‐fold faster depolymerization (Fig. 3B, Table 2).

The reduced F‐actin stability, the rapid depolymerization and the reduced polymerization rate of the p.R196H variant prompted us to investigate the polymerization defect further. We performed sedimentation experiments with increasing concentrations of pre‐polymerized WT and p.R196H F‐actin (Fig. 3C). We found less p.R196H actin at every concentration in the pellet fraction compared to WT actin. Applying a linear regression to the data revealed that the critical concentration of p.R196H actin (c_c_ = 0.66 ± 0.08 μm) is increased 5.5‐fold compared to WT actin (c_c_ = 0.12 ± 0.06 μm).

### Polymerization defects of p.R196H are maintained in the presence of WT actin

**Fig. 4:**
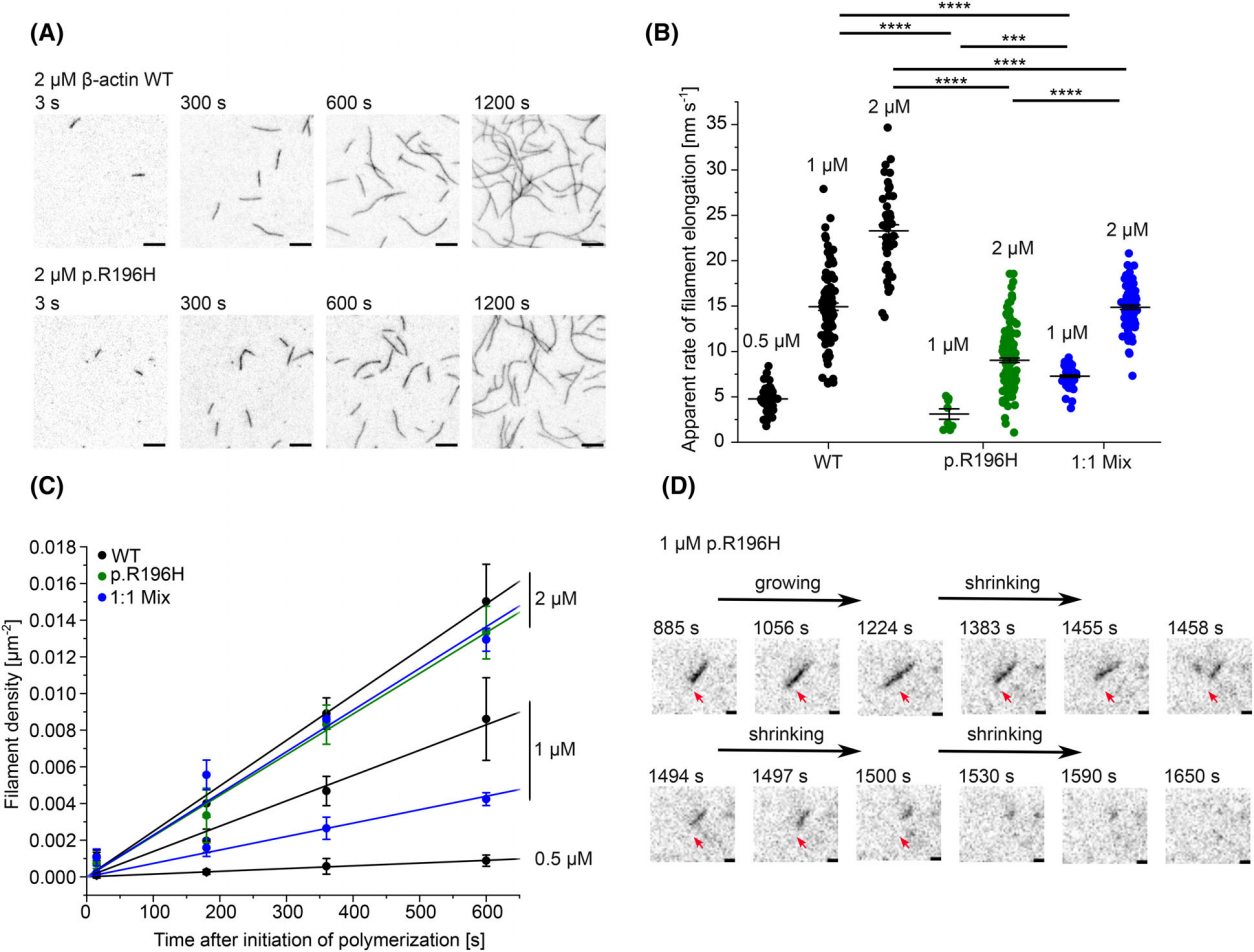
Analysis of the polymerization capacity of wild‐type and p.R196H β‐actin at different actin concentrations by total internal reflection fluorescence (TIRF) microscopy (A) Polymerization of p.R196H and wild‐type (WT) β‐actin (0.5, 1 and 2 μm; 10% ATTO‐655 labelled) was induced by salt‐shift, and the progression of the reaction was tracked by TIRF microscopy. Shown are representative micrographs at the indicated points in time from experiments performed with 2 μm p.R196H and WT actin (*N* = 3 for WT and *N* = 4 for p.R196H). Scale bar corresponds to 10 μm. (B) The elongation rates of individual filaments were determined by manual tracking of the elongating barbed ends of the filaments. Every data point represents an individual filament (WT (0.5 μm) = 55 filaments, WT (1 μm) = 97 filaments, WT (2 μm) = 47 filaments, p.R196H (1 μm) = 8 filaments, p.R196H (2 μm) = 159 filaments, Mix (1 μm) = 61 filaments and Mix (2 μm) = 90 filaments). Data are shown as the mean ± SEM. Statistical significance was determined by an unpaired two‐sample *t*‐test (*** ≙ *P* ≤ 0.001 and **** ≙ *P* ≤ 0.0001) (C) Nucleation efficiencies were calculated by determining the filament density [μm^−2^] over the time of the polymerization experiments. Linear regression was applied to the data to determine the apparent nucleation efficiency (*k*_nuc_) (Table 3). Data are shown as the mean ± SD (*N* = 3 for each condition with the exception of p.R196H (2 μm), where *N* = 4). (D) Exemplary p.R196H filament in polymerization experiment performed with 1 μm p.R196H actin (*N* = 3). The filament is loosely attached to the surface. One end of the filament (red arrow) shows slow polymerization followed by depolymerization. Scale bar corresponds to 1 μm.

Intrigued by the rapid depolymerization kinetics and the increased critical concentration of the p.R196H variant, we decided to investigate the effect of the R196H mutation on the polymerization capacity of actin in more detail. Therefore, we performed single‐filament polymerization studies using fluorescently labelled WT or p.R196H actin and total internal reflection fluorescence microscopy (TIRFM) (Fig. 4A,B). At a concentration of 1 μm, we observed efficient nucleation and elongation of WT filaments resulting in an average barbed‐end elongation rate of 14.9 ± 0.4 nm·s^−1^. In contrast, filaments were rarely observed in experiments conducted with p.R196H at the same concentration. Most of the observed p.R196H filaments were loosely attached to the surface, with one end exhibiting slow elongation (3.1 ± 0.6 nm·s^−1^), interspersed with periods of depolymerization (Fig. 4D). To increase the number of filaments observed, we increased the concentration of actin to 2 μm. Indeed, at 2 μm, we found significantly more p.R196H filaments in all experiments performed. The filaments showed an average elongation rate of 9.0 ± 0.2 nm·s^−1^, which is significantly lower than the elongation rate of WT filaments at 1 μm (14.9 ± 0.4 nm·s^−1^) and at 2 μm (23.3 ± 0.7 nm·s^−1^) (Fig. 4B). Since BWCFF is caused by heterozygous mutations, WT actin in patient cells produced by the unaffected allele may attenuate the observed molecular defects if it is capable of copolymerizing with p.R196H actin. Therefore, we repeated the TIRFM‐based experiments with 1 : 1 mixtures of p.R196H and WT actin. Experiments performed with a final concentration of 1 μm actin (0.5 μm p.R196H and 0.5 μm WT) produced sufficient filaments to determine the rate of elongation. Filaments in these experiments showed an average elongation rate of 7.3 ± 0.1 nm·s^−1^. This rate is significantly faster than the elongation rate of 0.5 μm pure WT actin (4.8 ± 0.2 nm·s^−1^) and significantly slower than the elongation rate of 1 μm pure WT actin (14.9 ± 0.4 nm·s^−1^). Therefore, p.R196H copolymerizes with WT actin into heterofilaments containing mutant and WT protein. The presence of WT actin attenuates the polymerization defect of p.R196H, but p.R196H still significantly affects the elongation rate of the heterofilaments. Increasing the actin concentration to 2 μm (1 μm p.R196H and 1 μm WT) also increased the observed elongation rate of the heterofilaments to 14.9 ± 0.2 nm·s^−1^, which is not significantly different to the elongation rate of 1 μm pure WT actin, but still slower than the observed elongation rate of 2 μm pure WT actin (23.3 ± 0.7 nm·s^−1^).

Every observed filament in the TIRFM‐based experiments is the result of a successful nucleation event. Therefore, we determined the filament density in WT and p.R196H experiments at distinct points in time to quantify the nucleation efficiency of WT and p.R196H actin at different concentrations (Fig. 4C). We observed a clear increase in the rate of filament nucleation (*k*_nuc_) of pure WT actin with increasing concentration (Fig. 4C, Table 3). We compared the nucleation efficiency of WT, p.R196H and the 1 : 1 mix of both at a final concentration of 2 μm actin and found only minimal differences at this concentration (Table 3). As previously stated, we rarely found p.R196H filaments at a concentration of 1 μm. These results point to a significant defect of filament nucleation at concentrations below 2 μm. As stated, we observed minimal differences in the nucleation efficiency between p.R196H actin and WT actin in TIRFM‐based experiments performed with 2 μm actin. However, in pyrene‐based bulk experiments, we detected a pronounced increase in *t*_lag_ for p.R196H compared to WT actin.

**Table 3:** Rates determined from TIRFM‐based studies using ATTO‐655‐labelled WT and p.R196H β‐actin.

	WT	p.R196H	1 : 1 mix
Elongation rate [nm·s^−1^]	4.8 ± 0.2 (0.5 μm) 14.9 ± 0.4 (1 μm) 23.3 ± 0.7 (2 μm)	n.d. 3.1 ± 0.6 (1 μm) 9.0 ± 0.2 (2 μm)	n.d. 7.3 ± 0.1 (1 μm) 14.9 ± 0.2 (2 μm)
Nucleation rate *k*_nuc_ [μm^−2^·s^−1^]	1.8 × 10^−6^ ± 0.3 × 10^−6^ (0.5 μm) 13.9 × 10^−6^ ± 3.2 × 10^−6^ (1 μm) 25.7 × 10^−6^ ± 3.9 × 10^−6^ (2 μm)	– – 22.4 × 10^−6^ ± 1.9 × 10^−6^ (2 μm)	– 7.3 × 10^−6^ ± 0.2 × 10^−6^ (1 μm) 22.2 × 10^−6^ ± 0.4 × 10^−6^ (2 μm)

### Arp2/3‐mediated generation of branched actin networks is less efficient for p.R196H

**Fig. 5:**
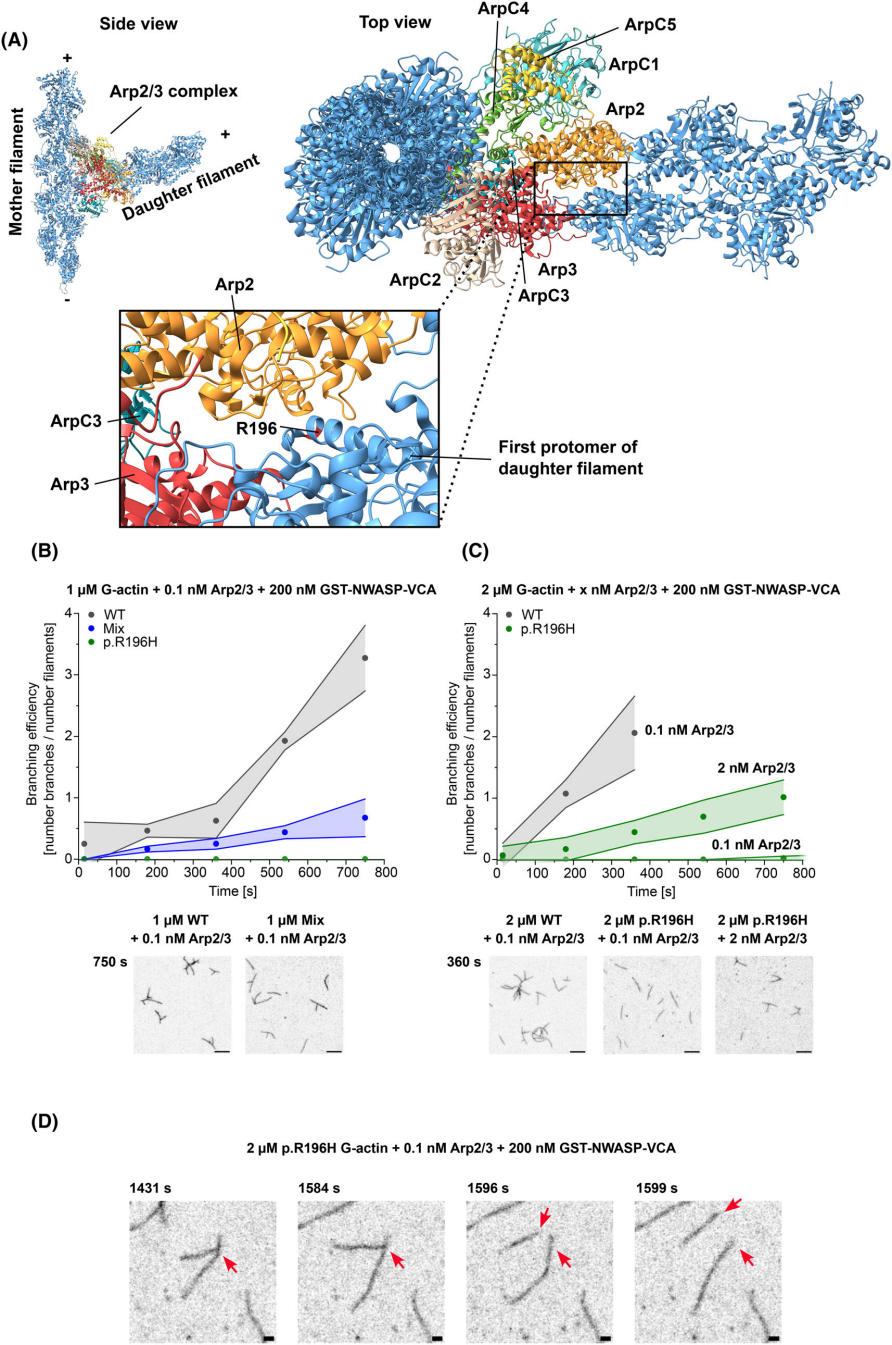
Analysis of the interaction of p.R196H and wild‐type filaments with the Arp2/3 complex by total internal reflection fluorescence (TIRF) microscopy (A) Structural model showing the actin–Arp2/3 branch junction complex (based on PDB‐ID: 7TPT [36]). The seven subunits of the Arp2/3 complex are differentially coloured. The zoom‐in shows the location of actin residue R196 at the interface between the initial protomer of the daughter filament and Arp2 of the Arp2/3 complex. Structure visualization was performed using chimerax [62]. (B, C) The formation of branched actin networks from p.R196H β‐actin, wildtype (WT) β‐actin and a 1 : 1 mixture (10% ATTO‐655 labelled) after salt‐induced polymerization in the presence of human Arp2/3 complex (+GST‐N‐WASP‐VCA) was investigated by TIRF microscopy. The branching efficiency (number of Arp2/3‐generated branches per initially linear filament) was determined for p.R196H and WT filaments at different actin and Arp2/3 complex concentrations. Plots show the determined branching efficiency over time. Data are shown as the mean ± SD of three individual experiments. Exemplary micrographs show Arp2/3‐generated branched actin structures at the indicated time after start of the experiment. Scale bar corresponds to 10 μm. (D) Time lapse showing the spontaneous debranching (red arrows) of a p.R196H branched actin filament at the indicated experimental conditions (*N* = 3). Scale bar corresponds to 1 μm.

The formation of branched actin structures absolutely requires the interaction of the actin filament with the Arp2/3 complex, one of the main regulators of actin nucleation *in vivo* [32]. The Arp2/3 subunits Arp2 and Arp3 show high structural homology to actin and form with subunits ArpC1, ArpC2, ArpC3, ArpC4 and ArpC5 a stable seven‐subunit complex that, after activation by a nucleation‐promoting factor (NPF), associates with the side of an existing filament and serves as a nucleation core for the addition of new actin protomers [33, 34, 35]. The newly added protomers intimately interact with Arp2 and Arp3, which form a stable dimer inside the complex structure [36]. Inspection of the high‐resolution structure of the actin branch junction reveals that residue R196 is located in regions important for the interaction of Arp2 with the first protomer of the newly formed daughter filament (Fig. 5A).

We analysed the interaction between p.R196H filaments and activated human Arp2/3 complex using TIRFM. When used in single‐filament TIRFM studies with 1 μm WT actin, 0.1 nm GST‐N‐WASP‐VCA‐activated human Arp2/3 complex was sufficient to induce the formation of branched actin structures (Fig. 5B). As already observed in our polymerization studies, 1 μm p.R196H actin is not sufficient to generate enough linear filaments for branching efficiency studies. Therefore, we performed experiments with 1 μm of a 1 : 1 mix of p.R196H and WT actin. We observed enough initially linear filaments to quantify the branching efficiency. We quantified the branching efficiency of Arp2/3 complex by counting the number of branching points divided by the number of observed initially linear filaments at different time points. Comparing the branching efficiency to the branching efficiency in experiments performed with 1 μm WT revealed a reduced efficiency for the heterofilaments, showing that the presence of p.R196H actin in the filament affects interaction with the Arp2/3 complex.

We increased the actin concentration to 2 μm p.R196H or WT actin to generate enough initial linear filaments in p.R196H samples to visualize the interaction of Arp2/3 complex with the pure mutant filaments (Fig. 5C). We found almost no branching in experiments performed with p.R196H and 0.1 nm Arp2/3 complex. It is interesting to note that in experiments with p.R196H filaments, we sometimes observed spontaneous debranching, a phenomenon that we never observed in experiments with WT actin (Fig. 5D). Increasing the Arp2/3 complex concentration 20‐fold in experiments with p.R196H still resulted in fewer observed branches compared to experiments with 2 μm WT actin and 0.1 nm Arp2/3 complex (Fig. 5C).

**Fig. 6:**
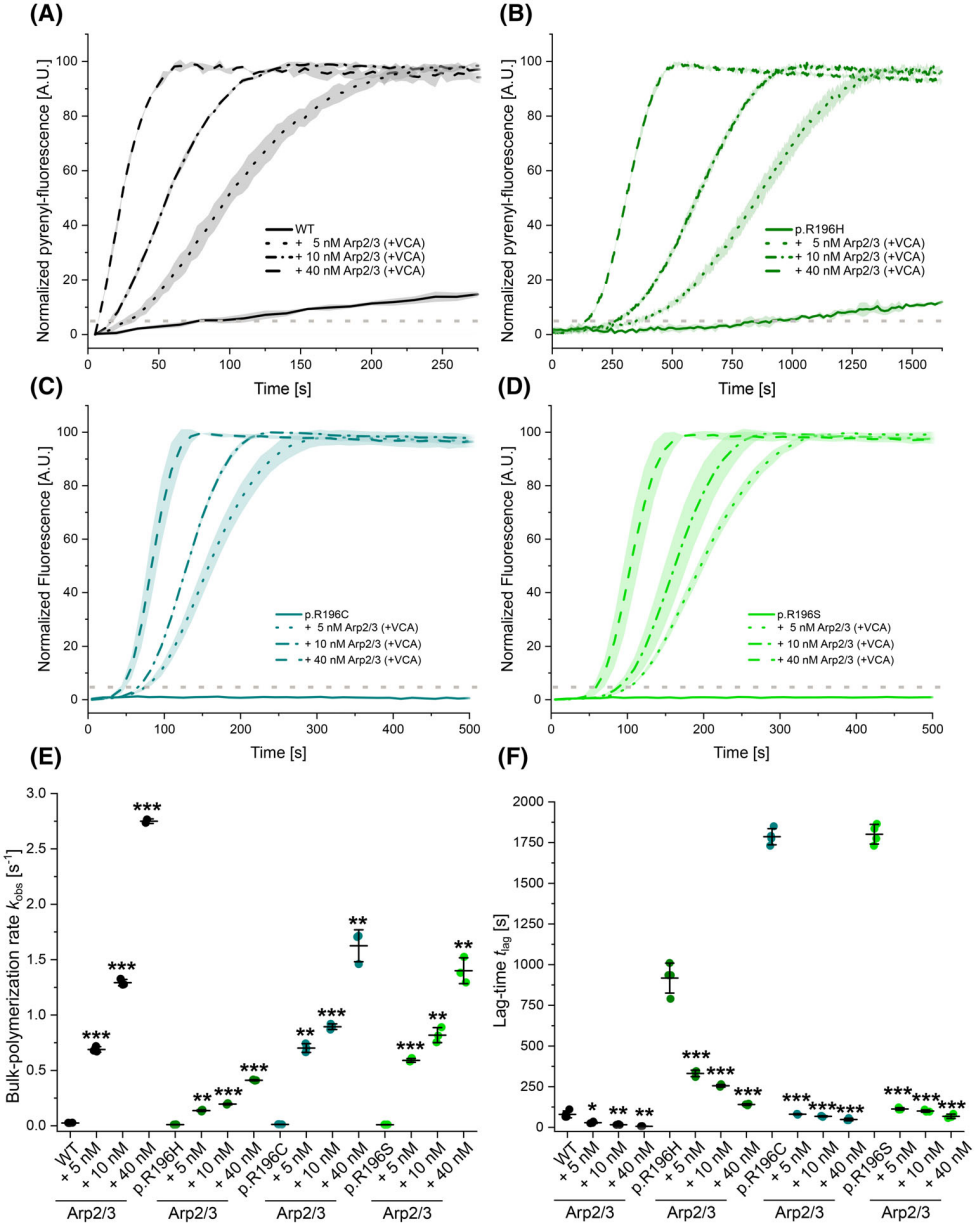
Analysis of the polymerization capacity of p.R196 variants and wildtype β‐actin in the presence of human Arp2/3 complex using bulk polymerization experiments. (A–D) Pyrene‐based polymerization experiment of 2 μm variant or wild‐type (WT) actin (5% pyrene labelled) in the absence and presence of increasing concentrations of Arp2/3 (+200 nm GST‐N‐WASP‐VCA). Traces are shown as the mean ± SD. Note the different *x*‐axis in (A), (B), (C) and (D). The dotted grey lines indicate 5% of the final pyrenyl fluorescence; the threshold that was used to determine the lag‐time *t*_lag_ in (F) (*N* = 4 for experiments in the absence of Arp2/3 and *N* = 3 for experiments in the presence of Arp2/3) (E) Bulk polymerization rates determined from the experiments shown in (A–D). Every data point corresponds to an individual polymerization experiment, as shown in (A–D). Data are shown as the mean ± SD. Individual values are summarized in Table 4. Statistical significance was determined by an unpaired two‐sample *t*‐test (** ≙ *P* ≤ 0.01, *** ≙ *P* ≤ 0.001) (*N* = 4 for experiments in the absence of Arp2/3 and *N* = 3 for experiments in the presence of Arp2/3). (F) Lag‐time (*t*_lag_) determined from pyrene‐based polymerization experiments. Every data point corresponds to an individual polymerization experiment. Data are shown as the mean ± SD. Individual values are summarized in Table 4. Statistical significance was determined by an unpaired two‐sample *t*‐test (* ≙ *P* ≤ 0.05, ** ≙ *P* ≤ 0.01 and *** ≙ *P* ≤ 0.001) (*N* = 4 for experiments in the absence of Arp2/3 and *N* = 3 for experiments in the presence of Arp2/3).

Since the TIRFM‐based assay does not allow an accurate quantitative assessment of branching efficiency under these conditions, we investigated the effect of higher Arp2/3 complex concentrations on WT and all three variants in bulk polymerization studies using pyrene‐labelled actin (Fig. 6A–D). Based on our previous results, we used 2 μm actin in our experiments to observe significant nucleation and elongation of p.R196H actin. The addition of VCA‐activated human Arp2/3 complex stimulated the polymerization of both variant and WT actin and affected both observed phases, as previously described [34]. The highest Arp2/3 concentration (40 nm) increased the *k*_obs_ of WT actin 109‐fold and reduced the *t*_lag_ by 12‐fold. In contrast, the highest Arp2/3 concentration had a milder effect on *k*_obs_ in experiments with p.R196H, resulting in only a 40‐fold increase in *k*_obs_. Accordingly, the effect on *t*_lag_ was also less pronounced compared to WT experiments (6.5‐fold decrease). Compared to the p.R196H variant, the *t*_lag_ and *k*_obs_ values of the pR196C and p.R196S variants were more significantly affected by the presence of the VCA‐activated Arp2/3 complex. However, even at the highest concentration of Arp2/3 complex used, the polymerization defects in these variants were not reduced to the levels observed with WT protein at the same concentration (Fig. 6E,F and Table 4).

**Table 4:** Rates determined from bulk polymerization studies with WT and variant β‐actin in the presence of Arp2/3 complex.

	Lag‐time *t*_lag_ [s]	Bulk‐polymerization rate *k*_obs_ [s^−1^]
WT	80.0 ± 21.2 29.0 ± 5.3 (5 nm Arp2/3) 16.3 ± 2.3 (10 nm Arp2/3) 6.8 ± 0.4 (40 nm Arp2/3)	0.0254 ± 0.0014 0.6891 ± 0.0252 (5 nm Arp2/3) 1.2922 ± 0.0289 (10 nm Arp2/3) 2.7497 ± 0.0208 (40 nm Arp2/3)
p.R196H	917.5 ± 92.1 331.7 ± 19.7 (5 nm Arp2/3) 255.7 ± 8.3 (10 nm Arp2/3) 140.3 ± 5.0 (40 nm Arp2/3)	0.0103 ± 0.0007 0.1369 ± 0.0076 (5 nm Arp2/3) 0.1959 ± 0.0064 (10 nm Arp2/3) 0.4112 ± 0.0048 (40 nm Arp2/3)
p.R196C	1786.3 ± 49.5 81.0 ± 3.5 (5 nm Arp2/3) 68.1 ± 3.4 (10 nm Arp2/3) 48.3 ± 7.5 (40 nm Arp2/3)	0.0124 ± 0.0005 0.7016 ± 0.0395 (5 nm Arp2/3) 0.8943 ± 0.0255 (10 nm Arp2/3) 1.6255 ± 0.1442 (40 nm Arp2/3)
p.R196S	1801.3 ± 60.5 113.3 ± 7.3 (5 nm Arp2/3) 100.3 ± 7.1 (10 nm Arp2/3) 66.0 ± 13.5 (40 nm Arp2/3)	0.0097 ± 0.0007 0.5904 ± 0.0171 (5 nm Arp2/3) 0.8186 ± 0.0680 (10 nm Arp2/3) 1.3999 ± 0.1163 (40 nm Arp2/3)

### Mutation R196H differentially affects interaction of F‐actin with myosin isoforms

**Fig. 7:**
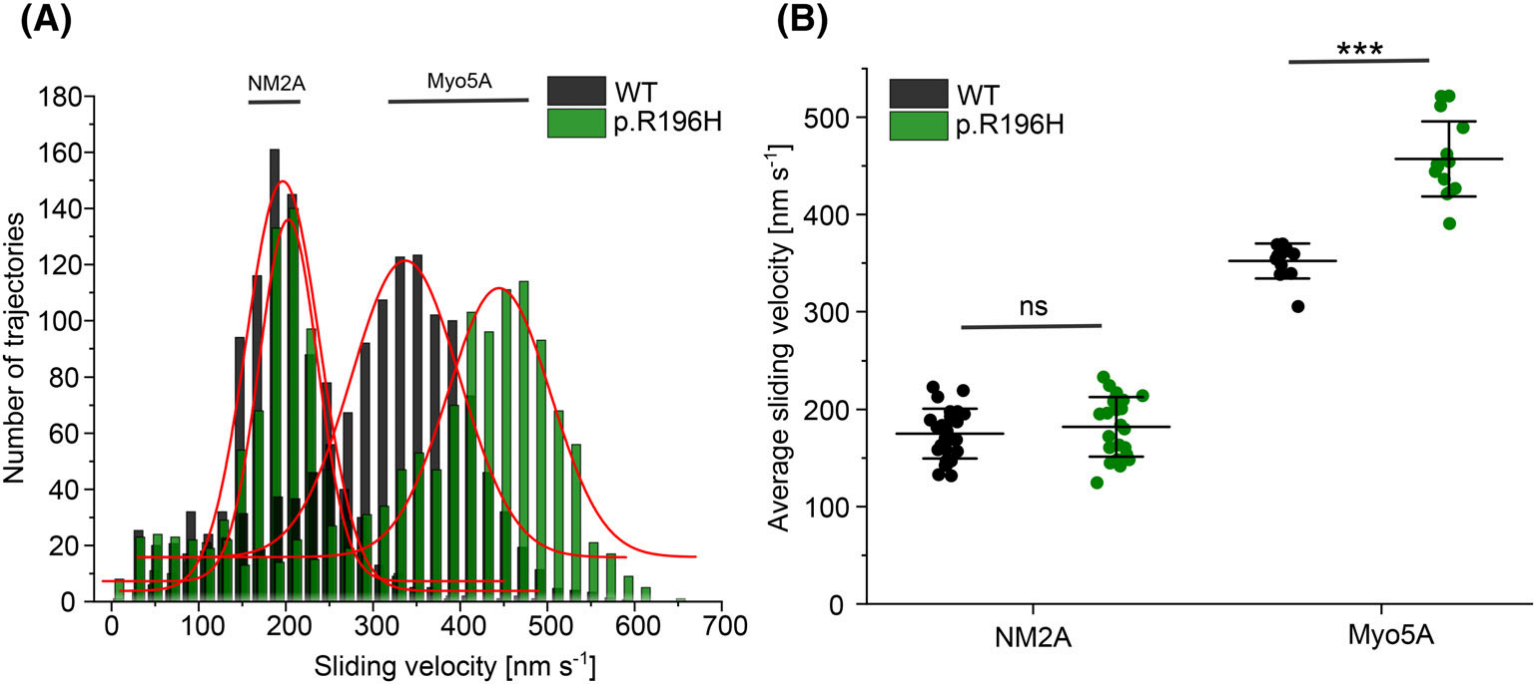
Analysis of the interaction of human non‐muscle myosin 2‐A (NM2A) and myosin‐5A (Myo5A) with p.R196H and wildtype β‐actin filaments. (A) The interaction of surface‐immobilized NM2A‐HMM and Myo5A‐HMM with TRITC‐phalloidin–stabilized WT β‐actin and p.R196H filaments was probed using the unloaded *in vitro* motility assay. Shown are representative velocity distributions obtained from recorded trajectories of WT and p.R196H filaments in a single experiment. A Gaussian fit (red line) was applied to the obtained velocity distributions in each experiment to determine the average sliding velocity of the respective filaments (*N* = 27 for WT filaments on NM2A, *N* = 23 for p.R196H filaments on NM2A, *N* = 12 for WT filaments on Myo5A and *N* = 15 for p.R196H filaments on Myo5A). (B) Secondary plot showing all measured sliding velocities. Each data point represents a single experiment (*N* = 27 for WT filaments on NM2A, *N* = 23 for p.R196H filaments on NM2A, *N* = 12 for WT filaments on Myo5A and *N* = 15 for p.R196H filaments on Myo5A). The average sliding velocity in each experiment was determined as described in (A). The mean ± SD of all performed experiments is shown. Statistical significance was determined by an unpaired two‐sample *t*‐test (ns ≙ *P* > 0.05 and *** ≙ *P* ≤ 0.001).

Residue R196 is buried inside the actin filament close to the filament axis and is therefore not directly involved in forming the actin–myosin interface. A previous study showed that a mutation in the hinge region of the actin protomer affects actomyosin interaction in a myosin isoform‐specific fashion, although the affected residue is buried inside the filament [37]. This effect was linked to mutation‐induced conformational changes in the actin filament that were maintained even in the presence of the filament‐stabilizer phalloidin. To address the possible effect of mutation‐induced conformational changes in the actin filament on the actomyosin complex, we analysed the interaction between p.R196H and WT filaments and two distinctly different myosin isoforms using the unloaded *in vitro* motility assay. We chose human myosin‐5A (Myo5A) and human non‐muscle myosin‐2A (NM2A) to reconstitute physiologically relevant actomyosin complexes (Fig. 7). TRITC phalloidin‐labelled cytoskeletal β‐actin filaments were moved by Myo5A with an average velocity of 352.2 ± 18.0 nm·s^−1^. In contrast, filaments formed by p.R196H were moved 1.3‐fold faster resulting in an average sliding velocity of 457.1 ± 38.6 nm·s^−1^. We observed no significant change in the velocity with which p.R196H and WT filaments were moved by NM2A, indicating a similar interaction with the stress fibre‐associated myosin (Fig. 7).

## Discussion

BWCFF is the best‐defined human syndrome associated with cytoskeletal β‐actin variants, and patients present with a relatively homogeneous phenotype [7, 8, 10, 15]. However, the link between the molecular perturbations of cytoskeletal actin dynamics caused by the mutated actin molecule and the phenotype of the patients remains largely unknown. *In vitro* characterization of the observed mutation‐induced defects is crucial for understanding the genotype–phenotype correlation, as it enables the dissection of individual steps in actin dynamics, both in the absence and in the presence of ABPs. The p.R196H variant is the most commonly observed variant associated with BWCFF, while p.R196C and p.R196S are less frequent but produce a similar phenotype. Affected patients exhibit characteristic craniofacial anomalies and neurodevelopmental disorders, which are thought to be related to neuronal migration defects [7, 8]. These variants therefore provide valuable insight into the molecular mechanisms underlying actinopathies.

Our study provides evidence that rapid protein degradation of p.R196 variants leading to functional haploinsufficiency is unlikely to be a contributing disease mechanism, as variant monomers are properly folded, stable and have nucleotide interactions comparable to WT β‐actin protein. Using TIRF microscopy‐based single‐filament studies with fluorescently labelled actin, we have shown that mutation R196H results in a reduced rate of barbed‐end elongation and a reduced rate of filament nucleation at low actin concentrations (< 2 μm). Both observed defects are attenuated in the presence of equimolar concentrations of WT actin, mimicking the expected outcome of heterozygosity in patients. Bulk polymerization studies additionally revealed reduced polymerization capacities of the variants p.R196C and p.R196S. Our observations suggest a disease mechanism involving the stable production of variant protomers and their efficient incorporation into actin filaments, thereby perturbing cytoskeletal filament dynamics and their interactions with ABPs. DSF and pyrene actin‐based bulk depolymerization experiments of variant and WT filaments provide further support for this interpretation, revealing the inherent and variant‐specific instability of mutant filaments and a compromised G‐ to F‐actin ratio *in vitro*. In an earlier study using lymphoblastoid cell lines from affected patients, no changes to the total cytoskeletal β‐actin and γ‐actin protein levels were observed, ruling out haploinsufficiency as a contributing disease mechanism, consistent with our *in vitro* characterization [8]. The same study describes an increased amount of F‐actin in fixed lymphoblastoid cells carrying p.R196H, an observation that cannot be easily explained by the positioning of the mutation in the actin filament structure and our experimental studies probing the actin dynamics of the variant *in vitro*. Further studies are therefore required to elucidate the contribution of the perturbed filament dynamics to the cellular phenotype observed in different cell types, in particular neurons and neuronal precursors, which are thought to be the most affected cell populations in the patients [7, 38].

A previous study used a cell‐free *in vitro* translation system to produce the variant p.R196C and showed that it folds as efficiently as WT protein [39]. Furthermore, the authors presented data that indicate a polymerization defect for the p.R196C variant, as filaments formed by the p.R196C variant *in vitro* are significantly shorter than WT filaments under the same conditions. These observations support our data, which revealed a reduced polymerization rate and faster depolymerization of p.R196C and p.R196S filaments. Therefore, it is reasonable to hypothesize that all BWCFF‐associated variants at position R196 disrupt the monomer–filament equilibrium by affecting critical intra‐protomer interactions in proximity to the filament axis. This interference is caused by introducing residues that either change the charge pattern (p.R196C and p.R196S) or are not sterically suited to maintain the H‐bond network with E253 and E237 (p.R196H). Furthermore, due to the close proximity of the affected residue to the interstrand contact formed between the neighbouring residues E195 and K113 of the opposite protomer, weakening of the interstrand contacts may contribute to the observed reduced filament stability (Fig. 1B).

R196 is located in SD3 of the actin protomer, adjacent to the helical axis of the actin filament, well away from the canonical interfaces for G‐ and F‐actin binding proteins [22]. However, this is not the case with the Arp2/3 complex, where the Arp2 and Arp3 subunits of the complex mimic a nucleation core for a daughter filament on the side of a mother filament. Due to the high structural similarity of the Arp2/3‐initiated daughter filament with the linear actin filament, we hypothesized that mutations that affect actin–actin interactions, as is the case for the p.R196 variants, impair the interaction of the variant protomers with the Arp2 and Arp3 subunits, reducing the efficiency of branch formation. In line with this hypothesis, we observed a decrease in branch formation efficiency and an apparent rise in branch instability in TIRFM‐based single‐filament studies and pyrene actin bulk polymerization experiments involving the p.R196H variant. While the resolution of the available branch–junction complex structures is not high enough to assign precise side‐chain conformations, it can be assumed that R196 in the initial protomer of the daughter filament is involved in the same H‐bond networks as it is in the linear filament and that the neighbouring residue E195 is involved in charge–charge interactions with residues of Arp2. Based on the similarity to linear actin filaments, we hypothesized that all variants at position R196 disrupt the interaction with the Arp2/3 complex. Our experiments performed with p.R196C and p.R196S showed that these variants do not perturb the interaction with the Arp2/3 complex as strongly as p.R196H. However, even at high concentrations of the Arp2/3 complex, the observed polymerization defects were not reduced to the levels seen with WT protein at the same concentration. These findings suggest that the substitution of R196 with a bulkier residue disrupts the interaction with the actin nucleator more significantly. To better understand the differential effects of these mutations on Arp2/3 complex interaction, higher‐resolution structures of the Arp2–protomer interface are needed.

Studies investigating the molecular consequences of the p.K118N/M variants in cytoskeletal γ‐actin came to the conclusion that impaired actin–Arp2/3 interactions contribute to non‐syndromic hearing loss [40, 41]. In the context of BWCFF, neuronal migration defects are suggested to be causative for the occurrence of pachygyria in the patients carrying p.R196 variants leading to developmental disabilities [7, 10]. The Arp2/3 complex is an essential component of the protein machinery that drives directed cell migration [32] and is important for human corticogenesis [42, 43]. Several steps in human corticogenesis are directly dependent on intact Arp2/3 complex function, including the morphogenesis and migration of radial glial and oligodendrocyte precursors [43, 44, 45] and the migration of the mature neuron [42]. Furthermore, mutations in a repressor of the Arp2/3 complex, α‐catenin, lead to pachygyria, thereby providing a link between misregulated Arp2/3 complex function and cortical malformations [46].

Various myosin isoforms were shown to be essential for neuronal function. Myo5A is highly abundant in the brain and its importance for neuronal function is well established [47]. Various Myo5A‐mediated transport processes contribute to synaptic plasticity [47], motor learning [48] and neurotransmitter recycling [49, 50]. NM2A is enriched in the vasculature of the brain, where it contributes to angiogenesis [51]. We observed an effect of the p.R196H variant on the interaction of Myo5A with the actin filament, as exemplified by a 1.3‐fold increase in filament sliding velocity on surface‐immobilized Myo5A‐HMM, whereas the variant did not affect the velocity of filaments moving on surface‐immobilized NM2A‐HMM. The mutated residue R196 is buried inside the actin filament, far away from the highly conserved actomyosin interface. Therefore, the introduced histidine residue is unlikely to directly affect the F‐actin–Myo5A interface. In addition, phalloidin further restricts the structural flexibility of the actin filament, potentially attenuating mutation‐induced changes in filament structure [30]. Structural studies of various cytoskeletal F‐actin–myosin complexes have revealed remarkable structural conservation of the actomyosin interface of different cytoskeletal motor complexes [52, 53]. However, the available actomyosin structures derived from cryogenic electron microscopy studies only resemble the average of an ensemble of possible conformations. Using transient phosphorescence anisotropy, a previous study showed that the conformational dynamics of F‐actin varies considerably between filaments decorated with skeletal muscle myosin‐2 or Myo5A, and that binding of the motors to the filament induces motor‐specific conformational changes in decorated and undecorated filament regions [54]. It is possible that the mutation R196H slightly shifts the conformational ensemble in a direction that is tolerated by NM2A but affects interaction of Myo5A with the filament. A comparable observation has been made with the actin mutant p.G146V, skeletal muscle myosin‐2 and Myo5A [37].

In conclusion, the BWCFF hotspot variants at position 196 in cytoskeletal β‐actin result in a molecular phenotype that is distinct from previously characterized mutations in cytoskeletal β‐actin. The mutations do not affect protein stability and abundance, unlike mutations associated with *ACTB*‐associated thrombocytopenia, which is a different syndrome from BWCFF [9, 17]. Moreover, the observed perturbations of actin–actin and actin–ABP interactions are significantly more pronounced than the consequences of mutations that have been summarized in a third group of disorders, referred to as unclassified *ACTB*‐associated non‐muscle actinopathies [18]. We, therefore, conclude that the disturbed actin dynamics resulting from the incorporation of the p.R196 variants into actin filaments, in conjunction with a variant‐specific reduction in the efficiency of the actin–Arp 2/3 interaction and isoform‐specific defects in the interaction of actin and myosin, are responsible for the cortical malformations and neurological developmental disorders observed in patients with the p.R196 hotspot variants.

## Materials and methods

### Plasmids

The coding sequence of human cytoskeletal β‐actin (Uniprot ID: P60709) was fused via a C‐terminal linker (ASR(GGS)_3_A) to a His_8_‐tagged thymosin‐β4 moiety (Uniprot ID: P62328) and was cloned into the multiple cloning site of the pFastBac‐dual vector under control of the polyhedrin promotor. The plasmids encoding the p.R196 variants were generated by site‐specific mutagenesis of the WT plasmid and verified by sequencing. The pFastBac1 vectors for the production of the HMM‐like constructs of human NM2A and human Myo5A were described previously [55]. The coding sequences of human myosin light chains MYL6a (Uniprot ID: P60660) and MYL12b (Uniprot ID: O14950) were cloned into the pFastBac dual vector [55]. Human calmodulin (Uniprot ID: P0DP23) was cloned into the pFastBac dual vector. The human Arp2/3 complex consisting of subunits Arp2 (Uniprot ID: P61160), Arp3 (Uniprot ID: P61158), ArpC1B (Uniprot ID: O15143), ArpC2 (Uniprot ID: O15144), ArpC3A (Uniprot ID: O15145), ArpC4 (Uniprot ID: P59998), ArpC5 (Uniprot ID: O15511) and a C‐terminal FLAG tag on ArpC3A was produced using the expression vector pBIG2abc, provided by Roberto Dominguez (University of Pennsylvania, Philadelphia, USA). The VCA domain of human N‐WASP (Uniprot ID: O00401) was cloned into pGEX‐6P‐2 vector. The cDNA‐encoding human thymosin‐β4 (Uniprot ID: P62328) was a gift from Taro Uyeda (Waseda University, Tokyo, Japan) and subcloned into the pET23(+) vector to generate a His_8_‐tagged construct.

The generation of recombinant bacmids and viruses for the production of proteins in the Sf9 system followed the Bac‐to‐Bac baculovirus expression system protocol (Thermo Fisher Scientific, Waltham, MA, USA).

### Protein production and purification

Recombinant human cytoskeletal β‐actin WT and the p.R196 variants were produced in the form of actin–thymosin‐β4 fusion proteins in the baculovirus/*S. frugiperda* (Sf9) insect cell expression system and purified following a protocol previously described for human cytoskeletal γ‐actin [25]. Myosin HMM‐like constructs were coproduced with human Myl6/Myl12b (NM2A‐HMM) or human calmodulin (Myo5A‐HMM) in Sf9 cells and purified as described previously [55, 56]. Production and purification of the human Arp2/3 complex followed the published protocol by Zimmet *et al*. [57].

The human GST‐N‐WASP‐VCA construct was produced in ArcticExpress (DE3) cells and purified as previously described [25].

Human thymosin‐β4‐His_8_ was produced in Rosetta2 cells. For a typical purification, cells from 1 L of expression culture were resuspended in 100 mL of lysis buffer (10 mm TRIS pH 7.8, 100 mm NaCl, 15 mm imidazole, 5 mm β‐mercaptoethanol, 1 mm PMSF, 100 μg·mL^−1^ TAME, 80 μg·mL^−1^ TPCK, 2 μg·mL^−1^ pepstatin and 5 μg·mL^−1^ leupeptin) supplemented with 250 μg·mL^−1^ lysozyme (from hen egg white; Merck KGaA, Darmstadt, Germany) and incubated on ice for 30 min. The cells were lysed by sonification and treated with DNase‐I (Roche, Basel, Switzerland) for 30 min on ice to remove bacterial DNA. The lysate was cleared by centrifugation at 35 000 ***g*** for 30 min and the cleared supernatant was subsequently added to 3 mL of Pure Cube™ NiNTA material (Cube Biotech, Monheimam Rhein, Germany) equilibrated with lysis buffer. The suspension was incubated for 2 h at 4 °C under constant rotation. The beads were washed with a 25‐fold excess of wash buffer (10 mm TRIS pH 7.8, 50 mm NaCl and 15 mm imidazole) and loaded onto a gravity‐flow column. The protein was eluted with 250 mm imidazole pH 7.5 in 3 mL fractions. The fractions containing the pure protein were pooled and diluted to a final concentration of 10 mm imidazole pH 7.5. The diluted sample was concentrated using a Vivaspin 20 concentrator (MWCO: 5000; Sartorius, Göttingen, Germany) to a concentration of at least 1 mm. The concentration was determined by SDS‐gel electrophoresis followed by densitometry at a ChemiDoc‐MP gel documentation system (Bio‐Rad Laboratories, Inc., Hercules, CA, USA) due to the absence of aromatic amino acids in thymosin‐β4. A fragment of the C2 domain of myosin‐binding protein C (MyBPC) of known size (17 kDa) and concentration was used as a standard. The final protein was snap frozen in liquid nitrogen and stored at −80 °C until used.

### DNase‐I inhibition assay, DSF and nucleotide exchange assay

DNase‐I inhibition assays were performed as previously described [17, 25].

DSF experiments with monomeric and filamentous WT and mutant actin followed protocols previously described [17, 25], with slight modifications. Briefly, for DSF experiments with G‐actin in the presence of thymosin‐β4 the G‐actin/thymosin‐β4 complex was formed by incubating equimolar amounts of both proteins on ice for 30 min prior to measurements. For DSF experiments with phalloidin‐stabilized actin filaments, filaments were incubated overnight at 4 °C with a 1.5‐fold molar excess of phalloidin. The thermal denaturation temperature *T*_M_ was determined from the peak of the first derivative of the melting curve. The FWHM correlates with the cooperativity of the observed melting process.

The rate of nucleotide dissociation for monomeric mutant and WT β‐actin was determined using ε‐ATP (Jena Bioscience, Jena, Germany) at a HiTech Scientific SF61 stopped‐flow system (TgK Scientific Limited, Bradford on Avon, UK), as previously described [25].

### TIRF microscopy‐based assays

Filament nucleation and elongation of WT and p.R196H actin were determined in TIRF microscopy‐based assays using freshly clarified protein. WT and p.R196H actin were labelled at cysteine‐374 with ATTO‐655 (ATTO‐TEC, Siegen, Germany) to visualize nucleation and elongation of actin filaments, as previously described [17, 25]. The glass coverslips used for the assembly of flow cells were cleaned and surfaces were chemically treated following previously described protocols [17, 25]. Actin polymerization was induced by diluting the G‐actin solution to a final concentration of 0.5, 1 or 2 μm (10% labelled actin) with 2× TIRF buffer (20 mm imidazole, 50 mm KCl, 1 mm MgCl_2_, 1 mm EGTA, 0.2 mm ATP, 15 mm glucose, 20 mm β‐ME, 0.25% methylcellulose, 0.1 mg·mL^−1^ glucose oxidase and 0.02 mg·mL^−1^ catalase). After mixing, the solutions were immediately flushed into flow cells and image acquisition was started. For experiments performed in the presence of VCA‐activated human Arp2/3 complex, VCA‐activated Arp2/3 complex was pre‐diluted in KMEI buffer (10 mm imidazole pH 7.4, 50 mm KCl, 1 mm MgCl_2_ and 1 mm EGTA) and diluted to the final concentration in TIRF buffer before addition of actin.

Image series were acquired at an Olympus IX83 inverted fluorescence microscope (Olympus, Hamburg, Germany) equipped with a 60×/1.49 NA PlanApo TIRF oil immersion objective and an Orca Flash 4.0 CMOS camera (Hamamatsu Photonics Deutschland GmbH, Herrsching, Germany). Image analysis was performed using imagej [58]. The elongation rates of individual actin filaments were determined by manually tracking individual filaments. To determine the branching efficiency in the presence of Arp2/3, the number of branching points was manually counted at several time points and divided by the number of initially linear filaments.

### Sedimentation experiments

Actin sedimentation assays were performed as described previously [25]. Briefly, actin was polymerized for 3 h at room temperature by diluting G‐actin into F‐buffer (10 mm TRIS pH 7.8, 100 mm KCl, 5 mm MgCl_2_, 0.5 mm EGTA, 0.1 mm DTT and 0.1 mm ATP) in 1.5 mL polypropylene centrifuge tubes (Beckman Coulter, Brea, CA, USA) suited for high‐speed centrifugation to final concentrations ranging from 0.75 to 10 μm. Samples were centrifuged at 136 000 ***g*** (30 min, 4 °C) and the resulting pellet and supernatant fraction were subjected to SDS/PAGE. The gels were imaged at a ChemiDoc‐MP gel documentation system (Bio‐Rad Laboratories). The amount of actin in the pellet and the supernatant was determined by densitometry in the image lab software (Bio‐Rad Laboratories). The concentration of F‐actin in each sample was plotted against the total actin concentration in each sample. A linear regression was fitted to the complete dataset, which yields the critical concentration of actin polymerization (c_c_) as the intercept with the *x*‐axis.

### Pyrene actin‐based bulk polymerization assays

The pyrene actin‐based bulk polymerization experiments in the absence and presence of VCA‐activated Arp2/3 complex were performed at a Synergy 4™ microplate reader (BioTek Instruments, Winooski, VT, USA) using the built‐in filter set (excitation: 340/30 nm and emission: 400/30 nm) at 25 °C, as previously described [25]. For dilution‐induced depolymerization experiments, 20 μm of pre‐polymerized pyrene‐labelled F‐actin was rapidly diluted to 0.1 μm by addition of G‐buffer (10 mm TRIS pH 7.8, 0.2 mm CaCl_2_ and 0.2 mm ATP).

Bulk polymerization rates were determined according to Doolittle *et al*. [59] by fitting a linear regression to the linear region around the time point of half‐maximal fluorescence. The lag‐time (*t*_lag_) of the reaction was defined as the time point at which the reaction reaches 5% of the final fluorescence signal. The rate of filament disassembly determined from dilution‐induced depolymerization experiments was calculated by fitting a monoexponential function to the obtained data, including a linear component that accounts for bleaching.

Labelling of WT and p.R196H actin at cysteine‐374 using *N*‐(1‐pyrene)iodoacetamide (Thermo Fisher Scientific) followed established protocols [60].

### Unloaded *in vitro* motility assay

Unloaded *in vitro* motility assays to analyse the interaction between WT and p.R196H actin filaments and NM2A‐HMM or Myo5A‐HMM were performed as previously described [17, 25, 55]. Briefly, flow cells for use in the *in vitro* motility assay were constructed by using nitrocellulose‐coated coverslips. Prior to the experiments, purified NM2A‐HMM was incubated with the GST‐tagged kinase domain (residues 1425–1776) of human smooth muscle myosin light‐chain kinase (Uniprot ID: A0A8I5KU53) at a molar ratio of 10 : 1 in 25 mm MOPS pH 7.3, 50 mm KCl, 5 mm MgCl_2_, 1 mm CaCl_2_, 0.2 μm calmodulin, 3 μm regulatory light chain (MYL12b), 3 μm essential light chain (MYL6a), 1 mm DTT and 1 mm ATP for 30 min at 30 °C. This is necessary as phosphorylation of the MYL12b is absolutely required for full activity of NM2A‐HMM. Myo5‐HMM was incubated with 2 μm human calmodulin and each buffer was supplemented with calmodulin to prevent calmodulin dissociation from Myo5A‐HMM during the experiments. An Olympus IX70 inverted fluorescence microscope (Olympus) equipped with a 60×/1.49 NA PlanApo oil immersion objective and an Orca Flash 4.0 CMOS camera (Hamamatsu Photonics Deutschland GmbH) was used for time‐lapse image acquisition. Experiments were performed at a 37 °C. The imagej Plugin wrmTrck [61] was used to determine the trajectories and corresponding velocities of the individual actin filaments.

### *In silico* analysis

Assessment and visualization of the potential implications of p.R196 variants for actin structure were performed using chimerax [62].

### Data analysis

Data analysis and graph plotting were performed with Origin 2023 (OriginLab Corporation, Northampton, MA, USA). Errors are given as standard deviation (SD) based on three independent experiments if not otherwise specified. The significance of the data was evaluated in Origin 2023 using a two‐sample *t*‐test (*P* > 0.05 ≙ ns, *P* ≤ 0.05 ≙ *, *P* ≤ 0.01 ≙ **, *P* ≤ 0.001 ≙ *** and *P* ≤ 0.0001 ≙ ****).

## Conflict of interest

The authors declare no conflict of interest.

## Author contributions

JNG purified proteins; JNG performed experiments; JNG and DJM analysed the data; JNG designed figures; JNG and DJM conceived and coordinated the study and wrote the manuscript; DJM was responsible for funding acquisition, supervision and project administration.

## Supporting information


**Fig. S1.** Visualization of variants p.R196C and p.R196S in the F‐actin structure.

## Data Availability

Unique reagents generated in this study are available from the lead contacts with a completed Materials Transfer Agreement.
