# Supplementary material for: Molecular mechanisms of hotspot variants in cytoskeletal β‐actin associated with Baraitser–Winter syndrome
Source: FEBS J. 2025 Feb 10;292(18):4898–917. doi: 10.1111/febs.70018 (PMC12443469; doi:10.1111/febs.70018)
Supplement: Supplementary file 1 — Fig. S1. Visualization of variants p.R196C and p.R196S in the F‐actin structure. [file FEBS-292-4898-s001.pdf]

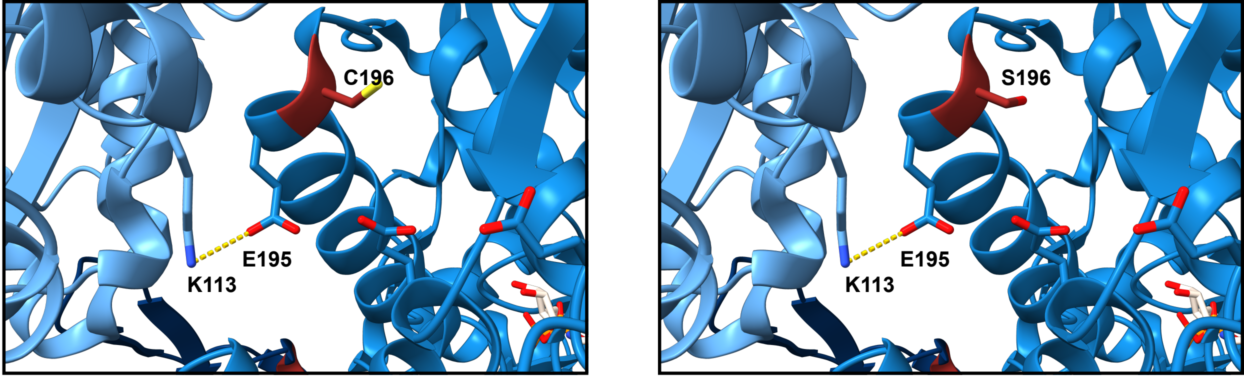

Supplementary Figure 1: Visualization of variants p.R196C and p.R196S in the F-actin structure. Structure visualization was performed using ChimeraX [62]
